# Editorial: Color change: neural and hormonal control of pigmentation

**DOI:** 10.3389/fendo.2024.1476470

**Published:** 2024-08-19

**Authors:** Maria Aparecida Visconti

**Affiliations:** Instituto de Biociências, Universidade de São Paulo, São Paulo, Brazil

**Keywords:** chromatophores, pigmentation and color, MCH, catecholamine, alpha-MSH

The pigment system, found in vertebrates, crustaceans, and cephalopods, related to diverse functions, is fundamental for the survival and adaptation of these organisms, enabling them to respond to both internal and external stimuli (Assis et al.).

For organisms to be able to present such varied colors, they must have molecules or structures that absorb light at certain wavelengths, and that emit at a wavelength complementary to that absorbed. These pigments are contained in specialized cells, chromatophores, present mainly in the skin, and can also be found in other regions of the body. These chromatophores in vertebrates originate from neural crest cells and have, in their differentiated state, dendrites that radiate from the cell body. Pigments are stored in organelles present in the cytoplasm. Many crustaceans, cephalopods and ectothermic vertebrates have different types of chromatophores, capable of adjusting the animal’s color in different situations. In contrast, birds and mammals have only one type of pigment cell, called a melanocyte (Zhao et al.).

The behavior of chromatophores is controlled by the nervous system, hormonal system, or both, with faster or slower changes. Several hormones and neurotransmitters are known to regulate chromatophore responses, inducing the aggregation of pigment granules perinuclearly, or their dispersal to dendritic projections (Assis et al., Shinohara et al., Yuan et al.).

In addition to rapid or physiological responses, these cells can change their number or size when the stimulus persists for a sufficiently long time, so-called morphological color changes, which also allow animals to adapt more efficiently to the environment.

There are basically five types of chromatophores, present in crustaceans and cephalopods and in ectothermic vertebrates. The chemical nature of pigments and the color they give to pigment cells were used to classify them.

Melanophores or melanocytes are the most widely distributed chromatophores and are responsible for much of the pigmentation in vertebrates. These cells produce two types of melanin: eumelanin (brown or black) or pheomelanin (yellow or red). Both have a dendritic appearance.

The erythrophore, reddish in color and dendritic in appearance, contain pigments such as carotenoids and pteridines. The xanthophores, which are yellowish in color, are also dendritic chromatophores. In general, they are smaller than melanophores and erythrophores, and contain carotenoids and pteridines, just as in erythrophore, however, in different proportions.

Leucophores also have a dendritic appearance, but their optical properties are entirely different. Instead of absorbing, they reflect light through purine granules found in their cytoplasm. Iridophores, metallic in color, have purines (guanine) arranged in the form of crystalline plates and reflect incident light. Both give the fish an iridescent, blue, silver, golden or white color, depending on the structural arrangement of these pigments. These colors are derived from at least one of two phenomena: interference and diffraction of light (Zhao et al.).

The combination and interaction between these chromatophores result in different chromatic patterns. In addition, hormones, and neurotransmitters control pigment migration in ectothermic vertebrates, of which α-MSH, MCH, catecholamines (both of humoral and nervous origin) and melatonin are undoubtedly the most important and well-known in terms of their function in hormonal regulation. of the pigment cell (Assis et al., Shinohara et al.).

The receptors for these compounds are members of the largest known superfamily of receptors, which are those coupled to the G protein. These receptors have seven transmembrane domains and intra and extracellular loops. The G protein is a heterotrimeric enzyme with three subunits: α, β and γ. The binding of an agonist to these receptors leads to the exchange of a GDP molecule for GTP and the dissociation of the α subunit from the βγ subunits. The α subunit, which has GTPase activity, is then free to activate membrane enzymes or ion channels, but in some cases the βγ subunits are the active subunits. The action of the effector ends by the hydrolysis of GTP bound to the α subunit, which allows its recombination with βγ.

Pigment dispersion is induced by α-MSH or catecholamines, when they bind to β adrenoceptors, and in both cases their receptors are coupled to a G protein that activates the adenylyl cyclase enzyme, leading to an increase in intracellular cAMP levels and PKA activation. Certain wavelengths also induce pigment dispersion in teleosts, but the mechanism of action is still poorly understood.

Pigment aggregation is induced by catecholamines, MCH and melatonin. The signaling pathways involve Gi/Go proteins, with a decrease in intracellular levels of cAMP, and/or Gq, with the participation of IP3, diacylglycerol and calcium, activating PKC (Zhao et al., Shinohara et al.).

Catecholamines can induce aggregation through two subtypes of adrenoceptors, α_1_ and α_2_, and the signaling pathways triggered by one or the other are different. Activation of α2 inhibits adenylyl cyclase, decreases cAMP levels and inactivates PKA. As for α_1_ adrenoceptors, they are coupled to the Gq protein, therefore there is activation of PKC and an increase in intracellular calcium levels.

For MCH, there is experimental evidence for two signaling pathways in teleost chromatophores. In some species there is a reduction in cAMP, therefore the receptor would be linked to the Gi protein. In others, the receptor would couple to the Gq protein, activating phospholipase C, generating IP3 and diacylglycerol, increasing intracellular calcium levels and activating Ca2+/calmodulin-dependent PKC. In teleosts, two receptors for MCH have been identified, so there is the possibility that they activate different G proteins in the same pigment cell.

The signaling pathways activated by melatonin were investigated in more detail in amphibians, in which melatonin binds to a high-affinity receptor and activates Gi/Go, inhibiting adenylyl cyclase, reducing cAMP and inhibiting PKA. There is another pathway that can be activated in the same receptor: Gβγ activates phosphoinositide-3-kinase (PI3-K), which activates a phosphodiesterase, resulting in a decrease in cAMP levels.

This pigment system, related to diverse functions, is fundamental for the survival and adaptation of different organisms, and allows them to respond to both internal and external stimuli. Chromatic adaptation as well as color patterns are phenomena that occur at the level of organisms, but depend on complex processes at the cellular and molecular level. Many of the topics covered present aspects that need to be more widely analyzed, which has been made possible by recent scientific advances and modern techniques in cell biology and physiology.

